# Galcanezumab for the Management of Migraine: A Systematic Review and Meta-Analysis of Randomized Placebo-Controlled Trials

**DOI:** 10.7759/cureus.11621

**Published:** 2020-11-22

**Authors:** Ahmed Abu-Zaid, Saud K AlBatati, Abdullah M AlHossan, Rayan A AlMatrody, Ayman AlGzi, Rayan A Al-Sharief, Faris M Alsobyani, Amena F Almubarak, Nadeen S Alatiyah

**Affiliations:** 1 Internal Medicine, College of Medicine, Alfaisal University, Riyadh, SAU; 2 Surgery, College of Medicine, Alfaisal University, Riyadh, SAU; 3 Neurology, College of Medicine, King Abdulaziz University, Rabigh, SAU; 4 Internal Medicine, College of Medicine, Arabian Gulf University, Manama, BHR; 5 Internal Medicine, College of Medicine, Dar Al Uloom University, Riyadh, SAU

**Keywords:** migraine, headache, calcitonin gene-related peptide, galcanezumab

## Abstract

Introduction

Migraine is a frequent neurological condition manifested by several episodes of headache. Calcitonin gene-related peptide (CGRP) has been shown to play a key role in the pathophysiology of migraine. Galcanezumab is a monoclonal antibody that binds CGRP and inhibits its action, without affecting the CGRP receptor. The aim of this study is to carry out a systematic review and meta-analysis of all randomized placebo-controlled trials that evaluated the efficacy of galcanezumab (120 mg or 240 mg) for the management of migraine.

Methods

We screened four databases (PubMed, SCOPUS, Embase, and Cochrane Central) from inception to October 10, 2020. Studies meeting the following criteria were included: (i) Patients: individuals with migraine, (ii) Intervention: galcanezumab at a dose of 120 mg or 240 mg, (iii) Comparator: placebo, (iv) Outcomes: prespecified efficacy and safety outcomes, and (v) Study design: randomized placebo-controlled trials. Efficacy outcomes included change in migraine headache days (MHDs), change in MHDs with acute medication use, patient global impression of severity (PGI-S) score, migraine-specific quality of life role function-restrictive domain (MSQ RF-R) score, and migraine disability assessment (MIDAS) score. Safety outcomes included frequency of injection-site pain, nasopharyngitis, and upper respiratory tract infection (URTI). Moreover, we used the Cochrane Collaboration's risk of bias tool to assess the risk of bias of the included studies. Review Manager Software, version 5.4.1, was used for statistical analysis. Mean difference and risk ratio with 95% confidence interval were used to analyze continuous and dichotomous outcomes, respectively. We used the fixed-effects and random-effects models to analyze homogeneous and heterogeneous data, respectively.

Results

A total of six studies comprising 4,023 patients were included in this systematic review and meta-analysis. When compared to placebo, both doses of galcanezumab were highly effective in decreasing MHDs (p<0.001), reducing MHDs with acute medication use (p<0.001), and improving the PGI-S score (p<0.001). On the other hand, MSQ RF-R and MIDAS scores were significantly enhanced only in the 240-mg dose group (p<0.001). With regard to side effects, the rates of injection-site pain and nasopharyngitis did not substantially differ between galcanezumab (inclusive of 120 mg and 240 mg) and placebo groups. Nonetheless, when compared to placebo, galcanezumab 120 mg, but not galcanezumab 240 mg, substantially correlated with a higher rate of URTI.

Conclusions

Galcanezumab is clinically safe and efficient for the management of migraine, and the use of a higher dose increases its efficacy. Future research directions should be geared toward determining the optimal dose of galcanezumab in the management of patients with migraine. Moreover, head-to-head comparative studies between galcanezumab and other related anti-CGRP receptor monoclonal antibodies are warranted.

## Introduction

Migraine is a frequent neurological condition manifested by several episodes of headache. These episodes are often accompanied by nausea, vomiting, and light hypersensitivity [1]. Migraine is categorized into two main types in accordance with the frequency of headaches: episodic migraine (<15 headache days per month) and chronic migraine (≥15 headache days per month) [2]. Migraine pathophysiology is not exactly known [1]. However, the accumulating body of research highlights a key role of calcitonin gene-related peptide (CGRP) in migraine pathophysiology [3,4]. This notion is supported by the observation that intravenous injection of CGRP results in spontaneous episodes of headache and migraine in migraineurs [3]. Moreover, blood levels of CGRPs are dramatically increased during migraine attacks [4].

Galcanezumab is a monoclonal antibody that binds CGRP and inhibits its action, without affecting the CGRP receptor [5,6]. Many clinical trials were performed investigating the efficacy of galcanezumab for the management of migraine. However, these clinical trials varied substantially with regard to the range of doses used. Moreover, till now, the proposed evidence from these clinical trials is contradictory. Therefore, the need for a comprehensive research that pools this evidence has become more required, which constituted the basic core of why we aimed to conduct this study to fill the literature gap. The objective of this study is to carry out a systematic review and meta-analysis of all randomized placebo-controlled trials that specifically evaluated the efficacy and safety of galcanezumab (120 mg or 240 mg) in patients with migraine.

## Materials and methods

Research protocol

This research was conducted in compliance with the Preferred Reporting Items for Systematic Reviews and Meta-Analyses (PRISMA) guidelines [7] and the Cochrane Handbook for Systematic Reviews of Interventions, Version 5.1.0 [8].

Search strategy

Four databases (PubMed, SCOPUS, Embase, and Cochrane Central) were screened from inception to October 5, 2020. The following search strategy was used in screening for relevant studies: (galcanezumab OR emgality OR LY2951742 OR LY-2951742 OR ajovy OR galcanezumab-gnlm OR aimovig) AND (migraine). There was no language restriction.

Eligibility criteria

Studies meeting the following criteria were included: (i) Patients: individuals with migraine, (ii) Intervention: galcanezumab at a dose of 120 mg or 240 mg, (iii) Comparator: placebo, (iv) Outcomes: prespecified efficacy (primary) and safety (secondary) outcomes, and (v) Study design: randomized placebo-controlled trials. Exclusion criteria included (i) patients with conditions other than migraine, (ii) interventional monoclonal antibodies other than galcanezumab, (iii) doses of galcanezumab other than 120 mg or 240 mg, (iv) animal trials, (v) nonrandomized human clinical trials, and (vi) studies not reporting the prespecified efficacy or safety outcomes.

Study selection

After screening of studies, duplicates were removed, and the remaining studies underwent a two-stage screening process. The first stage involved title and abstract screening. The second stage involved conducting full-text screening to exclude irrelevant trials. Moreover, we manually searched the reference lists of included studies to consider additional relevant studies. Two authors independently screened the studies and conflicts were resolved by a third author.

Risk of bias assessment

Cochrane Collaboration's risk of bias tool was used to assess the risk of bias of the included randomized placebo-controlled trials [9]. This risk tool consists of six domains: (i) sequence generation, (ii) allocation concealment, (iii) outcomes blinding, (iv) incomplete data, (v) selective reporting, and (vi) other bias. We scored each domain as unclear, low, or high risk. Two authors independently assessed the risk of bias, and conflicts were resolved by a third author.

Data extraction

The following three categories of data were collected: (i) baseline characteristics of the included studies, (ii) efficacy outcomes, and (iii) safety outcomes. Baseline characteristics of the included studies included first author, year of publication, national clinical trial (NCT) identifier, phase of clinical trial, type of migraine, study group, and sample size. Efficacy outcomes included change in monthly migraine headache days (MHDs), change in monthly MHDs with acute medication use, patient global impression of severity (PGI-S) score, migraine-specific quality of life role function-restrictive (MSQ RF-R) domain score, and migraine disability assessment (MIDAS) score. Safety outcomes included frequency of injection-site pain, nasopharyngitis, and upper respiratory tract infection (URTI). Several authors extracted the necessary data.

Data analysis

Review Manager Software Version 5.4.1 was used for statistical analysis. Mean difference (MD) and risk ratio (RR) with 95% confidence interval (95% CI) were used to analyze continuous and dichotomous outcomes, respectively. Fixed-effects and random-effects models were used to analyze homogenous and heterogeneous data, respectively. Statistical heterogeneity between studies was assessed by I-squared (I^2^) test and the p-value of heterogeneity. Statistical heterogeneity was determined when I^2^ measured >50% and p-value of heterogeneity measured <0.1. Sensitivity analysis using Cochrane's leave-one-out method was used to resolve heterogonous outcomes. For all outcomes, subgroup analysis according to the galcanezumab dose was conducted (120 mg/240 mg versus placebo).

## Results

Literature search

**Figure 1:**
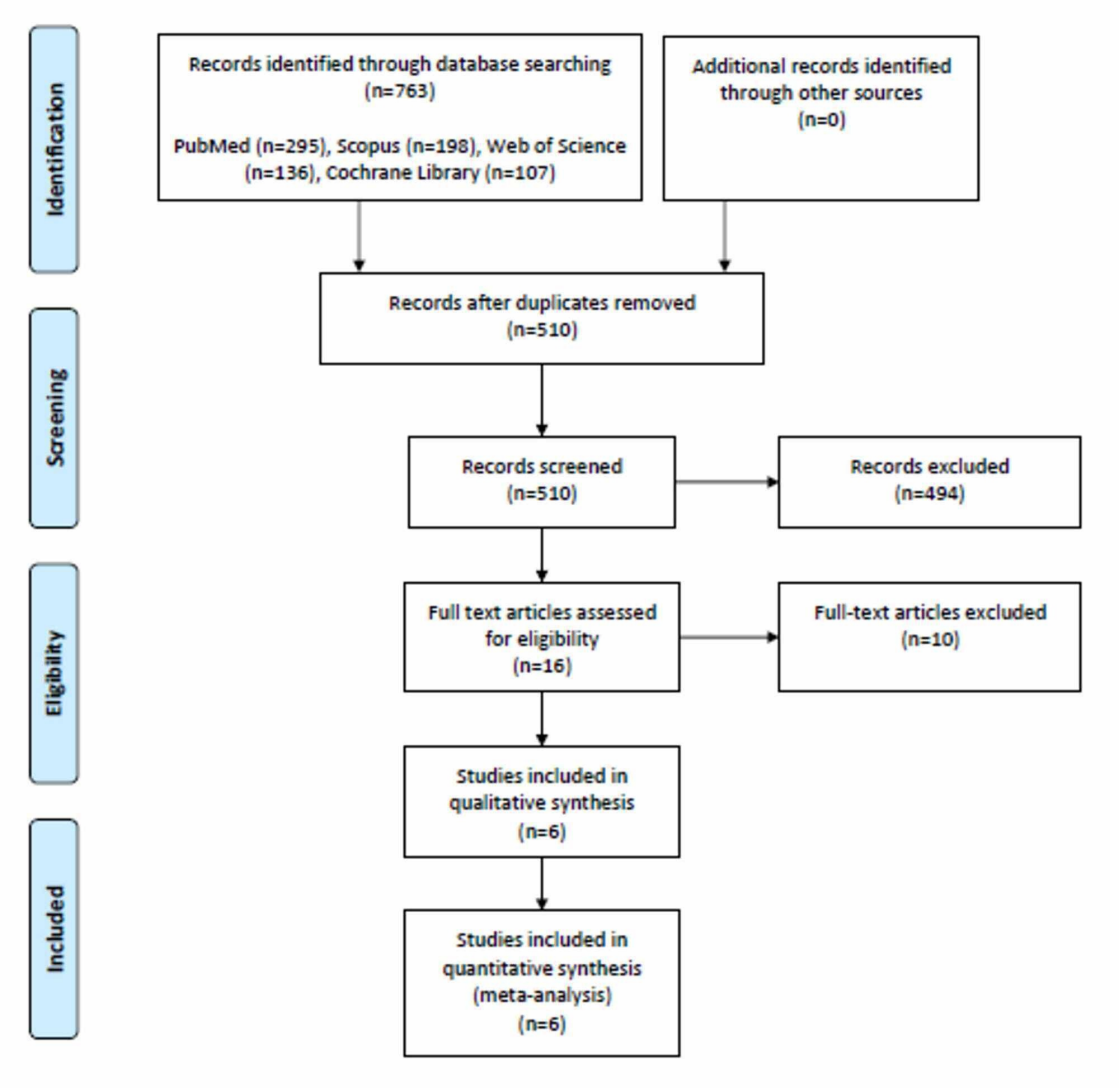
PRISMA flowchart. PRISMA, Preferred Reporting Items for Systematic Reviews and Meta-Analyses

Literature search yielded 510 studies. After screening, 490 studies were excluded because they did not match our inclusion criteria. Full-text screening of the remaining 20 studies resulted in an elimination of 14 studies that did not match our inclusion criteria. Finally, six studies comprising 4,023 patients were included in this systematic review and meta-analysis [5,10-14]. Figure 1 shows the PRISMA flowchart.

Galcanezumab and placebo were administered to 1,974 and 2,049 patients, respectively. The baseline characteristics of the included studies are depicted in Table 1.

**Table 1:** Baseline characteristics of the included studies. MHD, monthly headache days; MIDAS, migraine disability assessment; NCT, national clinical trial; NR, not reported; SD, standard deviation

Study identifier	NCT identifier	Phase	Condition	Study group	n	Female (%)	Migraine attacks per month, mean (SD)	MHDs per month with acute medication use, mean (SD)	MIDAS baseline score, mean (SD)
Stauffer et al., 2018 [5]	NCT02614183	3	Episodic migraine	Galcanezumab (120 mg)	213	85	5.6 (1.7)	7.4 (3.7)	32.9 (28.2)
				Galcanezumab (240 mg)	212	82.6	5.7 (1.8)	7.3 (3.3)	36.1 (27.8)
				Placebo	433	83.6	5.8 (1.7)	7.4 (3.5)	31.8 (27.3)
Detke et al., 2018 [14]	NCT02614261	3	Chronic migraine	Galcanezumab (120 mg)	278	85	NR	15.1 (6.3)	62.5 (49.5)
				Galcanezumab (240 mg)	277	82	NR	14.5 (6.3)	69.2 (64.1)
				Placebo	558	87	NR	15.5 (6.6)	68.7 (57.4)
Skljarevski et al., 2018 [10]	NCT02163993	2	Episodic migraine	Galcanezumab (120 mg)	70	84.6	4.6 (1.6)	NR	NR
				Placebo	137	79.6	4.7 (1.5)	NR	NR
Skljarevski et al., 2018 [11]	NCT02614196	3	Episodic migraine	Galcanezumab (120 mg)	231	85.3	5.54 (1.8)	7.47 (3.3)	30.9 (27.9)
				Galcanezumab (240 mg)	231	85.7	5.66 (1.8)	7.47 (3.3)	32.8 (28.8)
				Placebo	461	85.3	5. 7 (1.8)	7.6 (3.4)	34.3 (31.0)
Sakai et al., 2020 [12]	NCT02959177	2	Episodic migraine	Galcanezumab (120 mg)	115	82.6	5.6 (1.7)	7.3 (2.9)	14.8 (18.1)
				Galcanezumab (240 mg)	114	84.2	5.5 (1.8)	7.8 (3.0)	13.7 (13.9)
				Placebo	230	85.2	5.5 (1.7)	7.4 (3.0)	15.8 (19.3)
Mulleners et al., 2020 [13]	NCT03559257	3	Episodic migraine and chronic migraine	Galcanezumab (120 mg)	232	84	NR	12.3 (6)	50.9 (46)
				Placebo	230	88	NR	12.4 (6)	51 (45.5)

Results of risk of bias assessment

**Figure 2:**
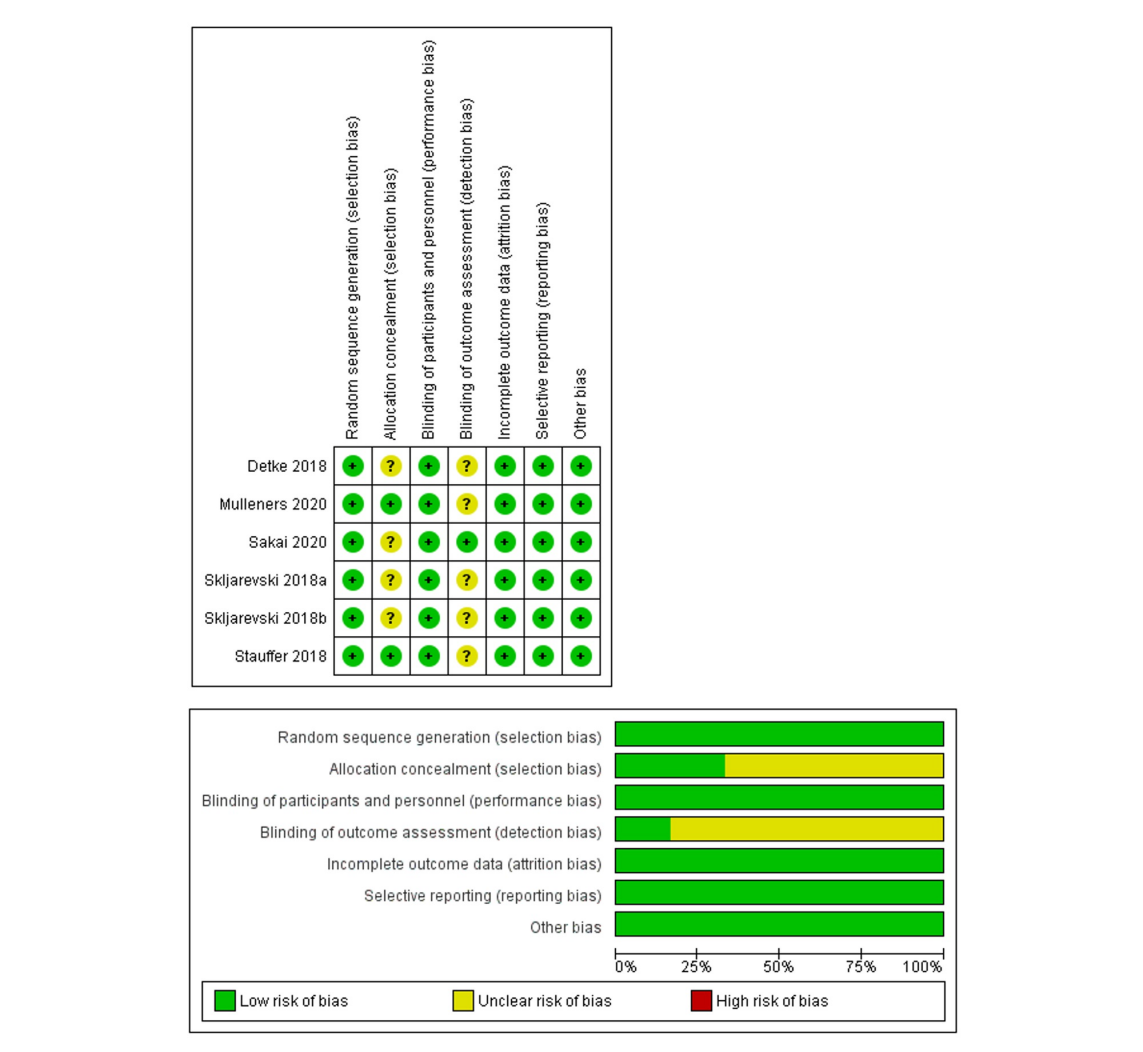
Risk of bias summary and graph.

All studies showed low risk of bias for the domains of random sequence generation, blinding of participants and personnel, incomplete outcome data, and selective reporting. Inadequate details were provided for allocation concealment and blinding of outcome assessments in some studies, and hence these domains were scored as unclear risk. Overall, all included studies revealed low-to-moderate risk of bias. Figure 2 shows the risk of bias summary and graph.

Efficacy outcome: overall mean change from baseline in the number of monthly MHDs

**Figure 3:**
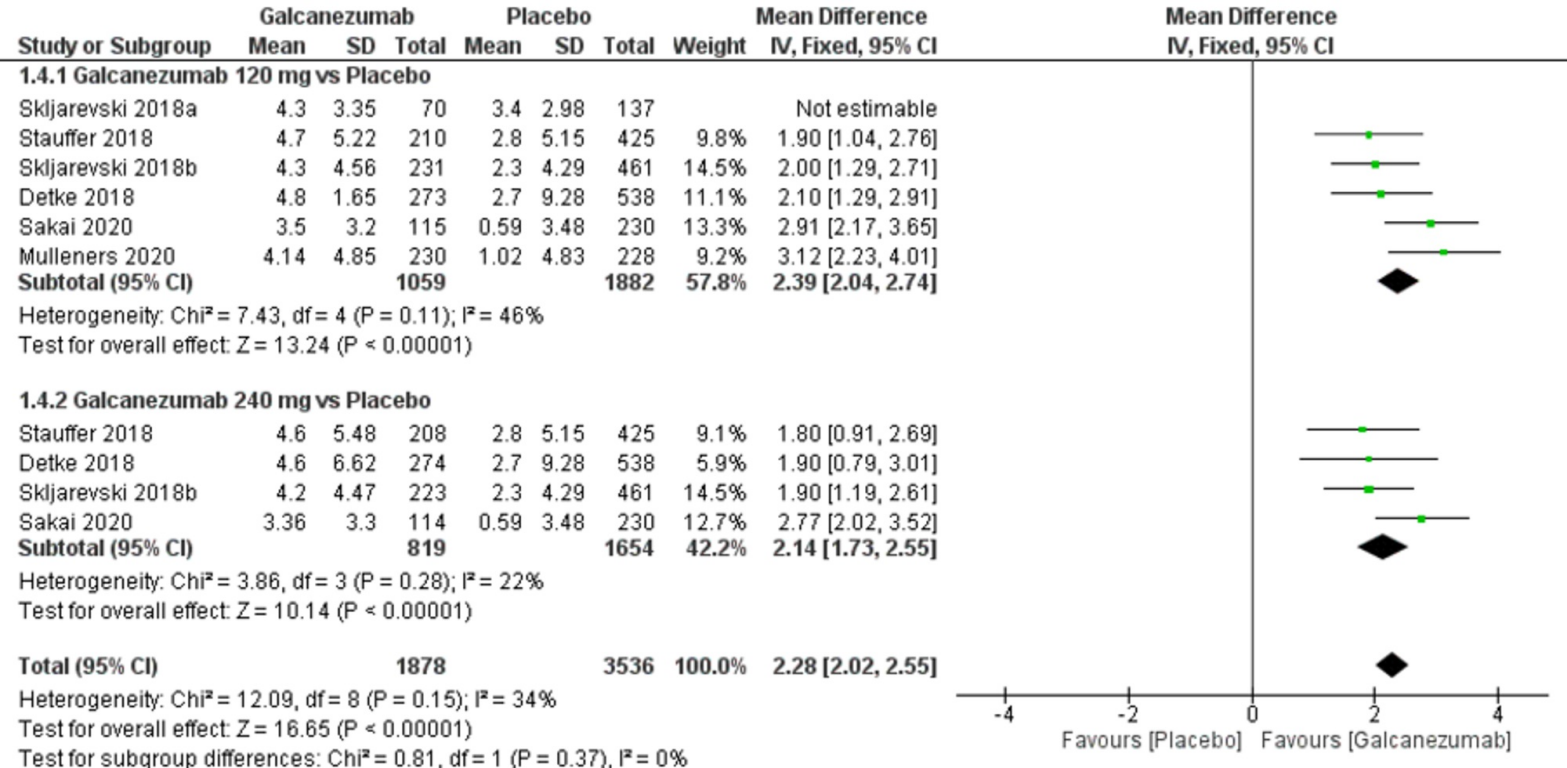
Forest plot showing the change in monthly migraine headache days between galcanezumab and placebo groups.

The overall effect size significantly favored galcanezumab over placebo (MD=2.28; 95% CI [2.02, 2.55]; p<0.001). Pooled results were homogeneous (I^2^=34%; p=0.15), and the fixed-effects model was used (Figure 3). Subgroup analysis was performed according to the galcanezumab dose. For galcanezumab 120 mg versus placebo, the overall effect size significantly favored the galcanezumab group (MD=2.39; 95% CI: [2.04, 2.74]; p<0.001). Pooled results were homogeneous (I^2^=46%; p=0.11). For galcanezumab 240 mg versus placebo, the overall effect size significantly favored the galcanezumab group (MD=2.14; 95% CI [1.73, 2.55]; p<0.001). Pooled results were homogeneous (I^2^=22%; p=0.28).

Efficacy outcome: overall mean change from baseline in the number of monthly MHDs with acute medication use

**Figure 4:**
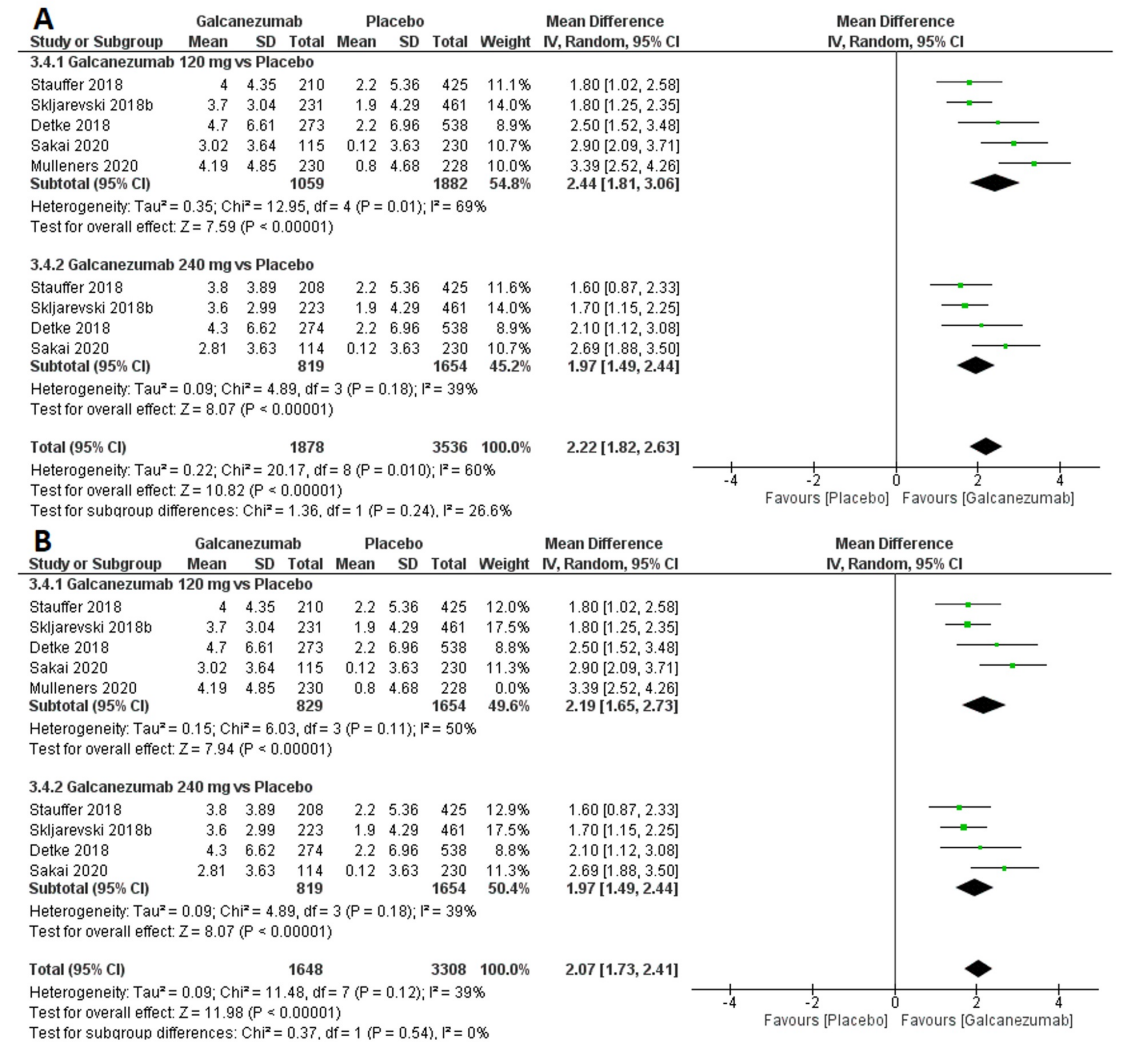
Forest plot showing the change in monthly migraine headache days with acute medication use between galcanezumab and placebo groups before (A) and after (B) sensitivity analysis using the leave-one-out method.

The overall effect size significantly favored galcanezumab over placebo (MD=2.22; 95% CI [1.82, 2.63]; p<0.001). Pooled results were heterogeneous (I^2^=60%; p=0.010), and the random-effects model was used (Figure 4). Subgroup analysis was performed according to the galcanezumab dose. For galcanezumab 120 mg versus placebo, the overall effect size significantly favored the galcanezumab group (MD=2.44; 95% CI [1.81, 3.06]; p<0.001). Pooled results were heterogeneous (I^2^=69%; p=0.01). Heterogeneity was best resolved (I^2^=50%; p=0.11) by omitting Mulleners et al.’ study [13], and the overall effect size still significantly favored the galcanezumab group (MD=2.19; 95% CI [1.65, 2.73]; p<0.001). For galcanezumab 240 mg versus placebo, the overall effect size significantly favored the galcanezumab group (MD=1.97; 95% CI [1.49, 2.44]; p<0.001). Pooled results were homogeneous (I²=39%; p=0.18).

Efficacy outcome: PGI-S score

**Figure 5:**
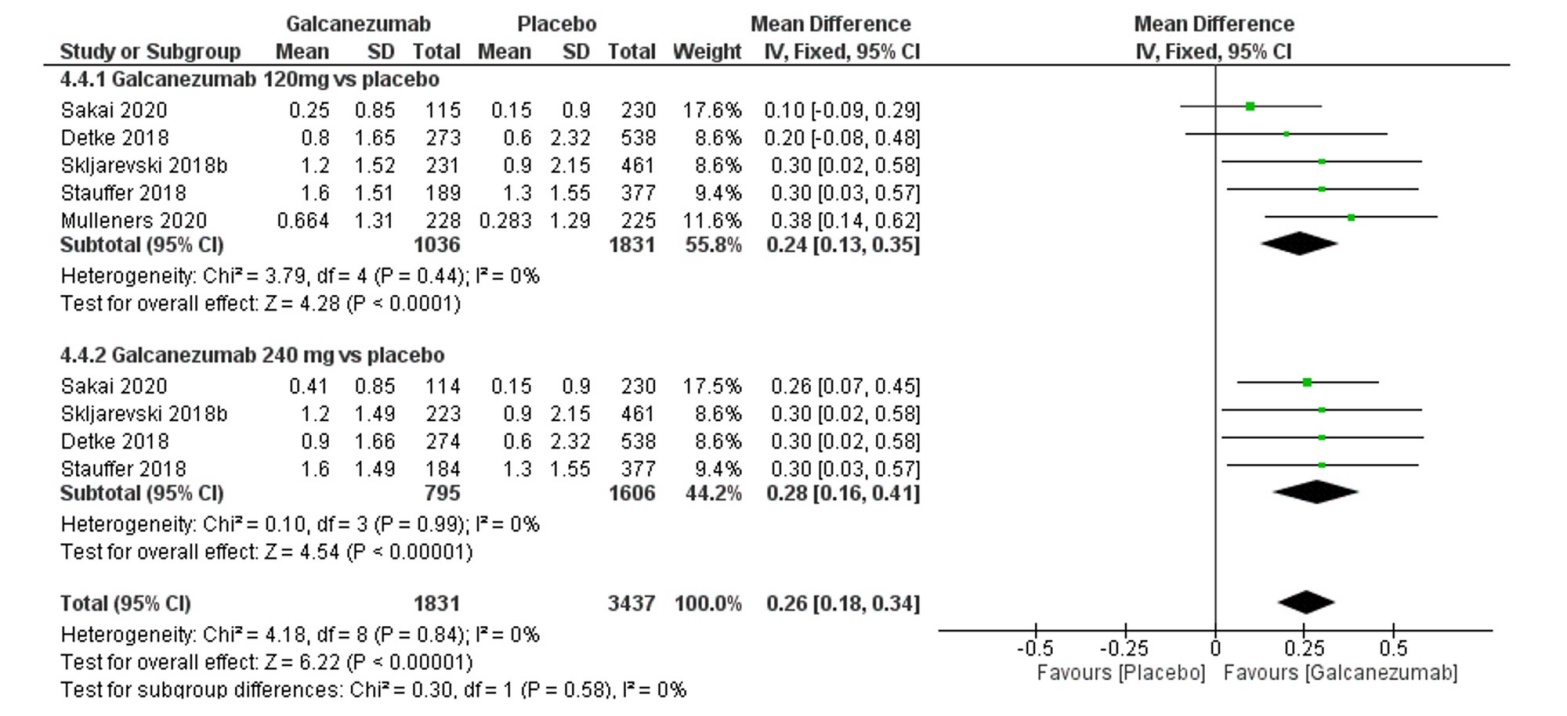
Forest plot showing PGI-S between galcanezumab and placebo groups. PGI-S, global impression score of severity

The overall effect size significantly favored galcanezumab over placebo (MD=0.26; 95% CI [0.18, 0.34]; p<0.001). Pooled results were homogenous (I^2^=0%; p=0.84), and the fixed-effects model was used (Figure 5). Subgroup analysis was performed according to the galcanezumab dose. For galcanezumab 120 mg versus placebo, the overall effect size significantly favored the galcanezumab group (MD=0.24; 95% CI [0.13, 0.35]; p<0.001). Pooled results were homogeneous (I^2^=0%; p=0.44). For galcanezumab 240 mg versus placebo, the overall effect size significantly favored the galcanezumab group (0.28 [0.16, 0.41]; p<0.001). Pooled results were homogeneous (I^2^=0%; p=0.99).

Efficacy outcome: MSQ RF-R score

**Figure 6:**
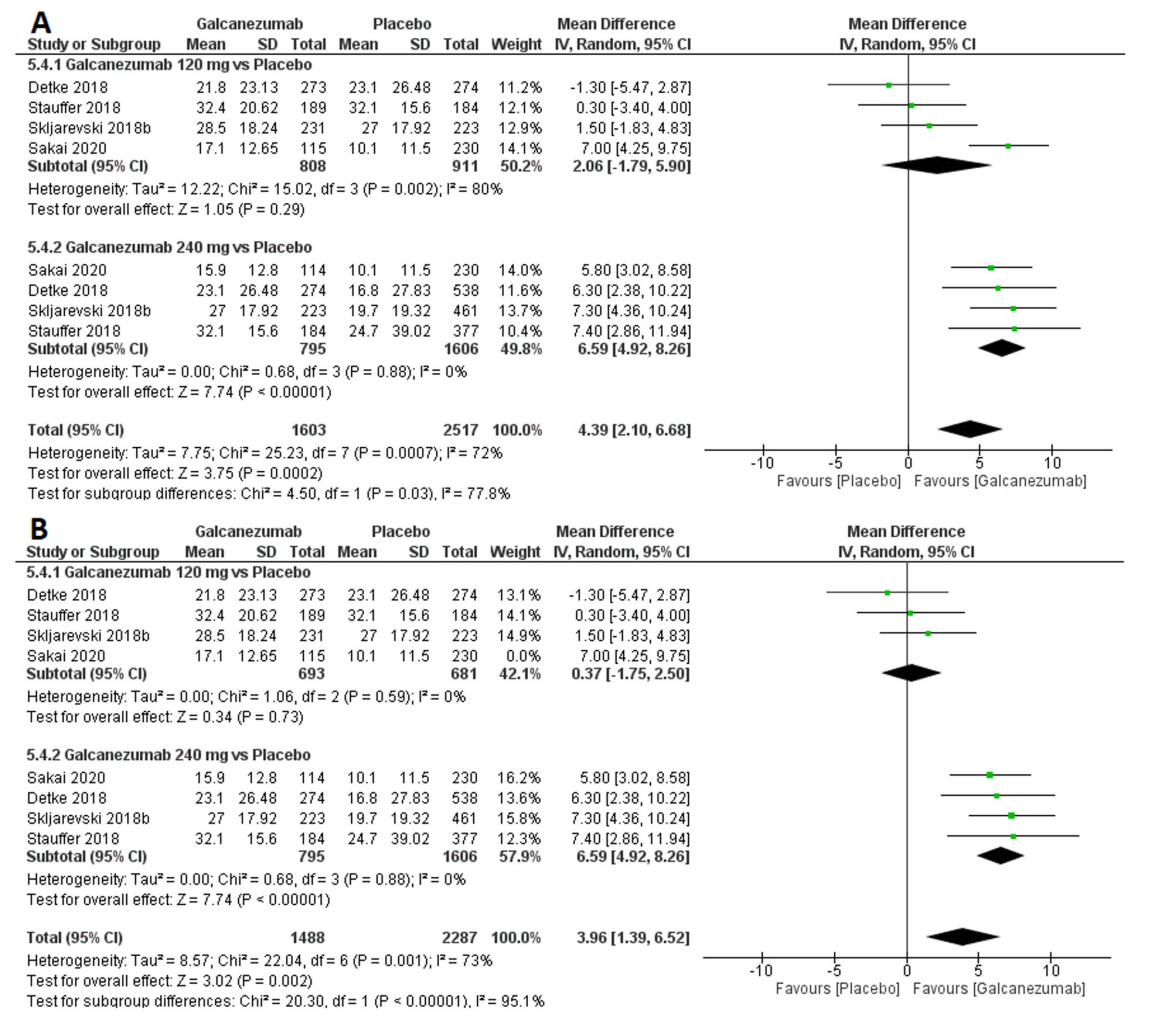
Forest plot showing the MSQ RF-R score between galcanezumab and placebo groups before (A) and after (B) sensitivity analysis using the leave-one-out method. MSQ RF-R, migraine-specific quality of life questionnaire role function-restrictive

The overall effect size significantly favored galcanezumab over placebo (MD=4.39; 95% CI [2.10, 6.68]; p<0.001). Pooled results were heterogeneous (I^2^=72%; p<0.001), and the random-effects model was used (Figure 6). Subgroup analysis was performed according to the galcanezumab dose. For galcanezumab 120 mg versus placebo, the overall effect size did not significantly differ between both groups (MD=2.06; 95% CI [-1.79, 5.90]; p=0.32). Pooled results were heterogeneous (I^2^=80%; p=0.002). Heterogeneity was best resolved (I^2^=0%; p=0.53) by omitting Sakai et al.’s study [12], and the overall effect size still did not favor any group (MD=0.37; 95% CI [-1.75, 2.50]; p=0.73). For galcanezumab 240 mg versus placebo, the overall effect size significantly favored the galcanezumab group (MD=6.59; 95% CI [4.92, 8.26]; p<0.001). Pooled results were homogeneous (I^2^=0%; p=0.88).

Efficacy outcome: MIDAS score

**Figure 7:**
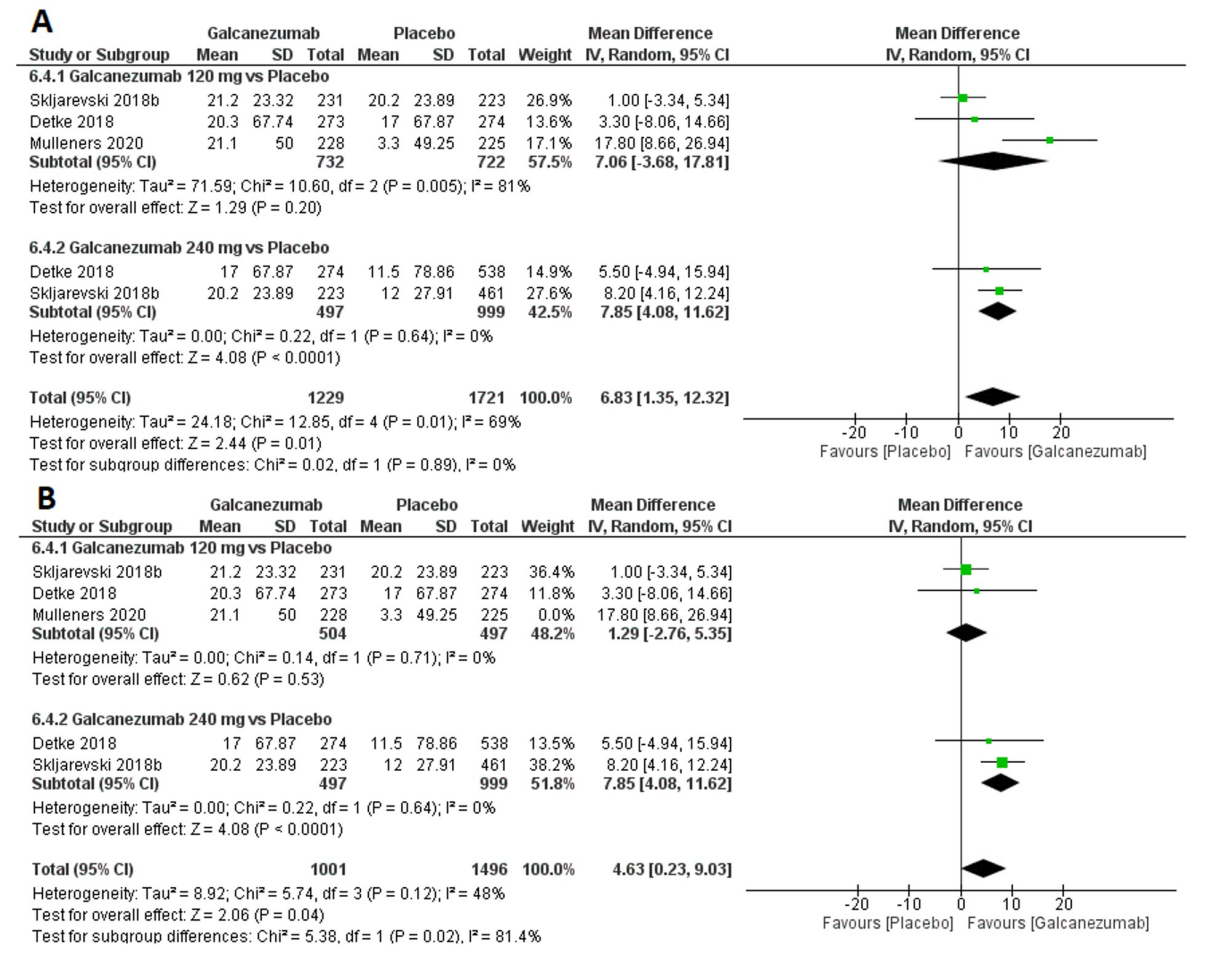
Forest plot showing MIDAS between galcanezumab and placebo groups before (A) and after (B) sensitivity analysis using the leave-one-out method. MIDAS, migraine disability assessment score

The overall effect size significantly favored galcanezumab over placebo (MD=6.83; 95% CI [1.35, 12.32]; p<0.001). Pooled results were heterogeneous (I^2^=69%; p=0.01), and the random-effects model was used (Figure 7). Subgroup analysis was performed according to the galcanezumab dose. For galcanezumab 120 mg versus placebo, the overall effect size significantly favored the galcanezumab group (MD=7.06; 95% CI [-3.68, 17.81]; p=0.20). Pooled results were heterogeneous (I^2^=81%; p=0.005). Heterogeneity was best resolved (I^2^=0%; p=0.71) by omitting Mulleners et al.’s study [13], and the overall effect size did not favor any group (MD=1.29; 95% CI [-2.76, 5.35]; p=0.53). For galcanezumab 240 mg versus placebo, the overall effect size significantly favored the galcanezumab group (MD=7.85; 95% CI [4.08, 11.62]; p<0.001). Pooled results were homogeneous (I^2^=0%; p=0.64).

Safety outcome: injection-site pain

**Figure 8:**
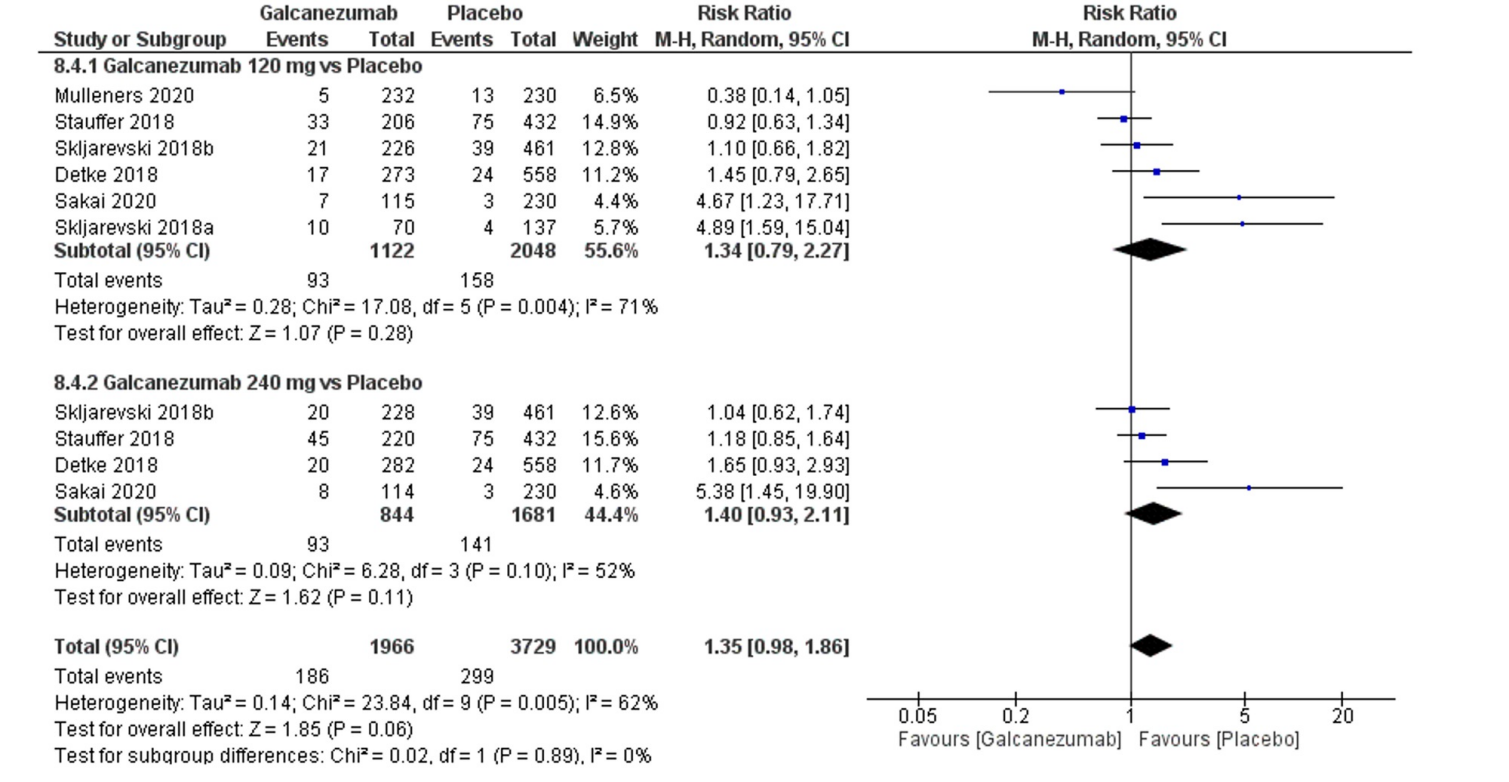
Forest plot showing the rate of injection-site pain between galcanezumab and placebo groups.

The overall effect size did not show a significant difference between both groups (RR=1.35; 95% CI [0.98, 1.86]; p=0.06) Pooled results were heterogeneous (I^2^=62%; p=0.005), and the random-effects model was used (Figure 8). Subgroup analysis was performed according to the galcanezumab dose. For galcanezumab 120 mg versus placebo, the overall effect size significantly did not differ between both groups (RR=1.34; 95% CI [0.79, 2.27]; p=0.28). Pooled results were heterogeneous (I^2^=71%; p=0.004). Heterogeneity could not be resolved by performing leave-one-out method. For galcanezumab 240 mg versus placebo, the overall effect size did not significantly differ between both groups (RR=1.40; 95% CI [0.93, 2.11]; p=0.11). Pooled results were homogeneous (I^2^=52%; p=0.10).

Safety outcome: nasopharyngitis

**Figure 9:**
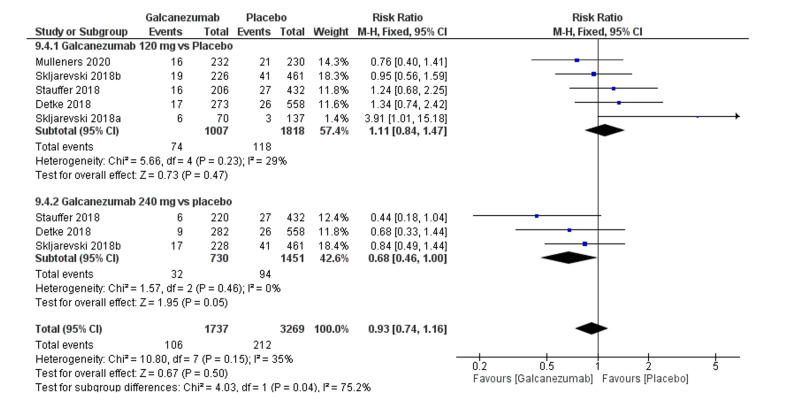
Forest plot showing the rate of nasopharyngitis between galcanezumab and placebo groups.

The overall effect size did not show a significant difference between both groups (MD=0.93; 95% CI [0.74, 1.16]; p=0.5). Pooled results were homogenous (I^2^=35%; p=0.15), and the fixed-effects model was used (Figure 9). Subgroup analysis was performed according to the galcanezumab dose. For galcanezumab 120 mg versus placebo, the overall effect size did not significantly differ between both groups (RR=1.11; 95% CI [0.84, 1.47]; p=0.47). Pooled results were homogeneous (I^2^=29%; p=0.23). For galcanezumab 240 mg versus placebo, the overall effect size did not significantly differ between both groups (RR=0.68; 95% CI [0.46, 1.00]; p=0.05). Pooled results were homogeneous (I^2^=53%; p=0.53).

Safety outcome: URTI

**Figure 10:**
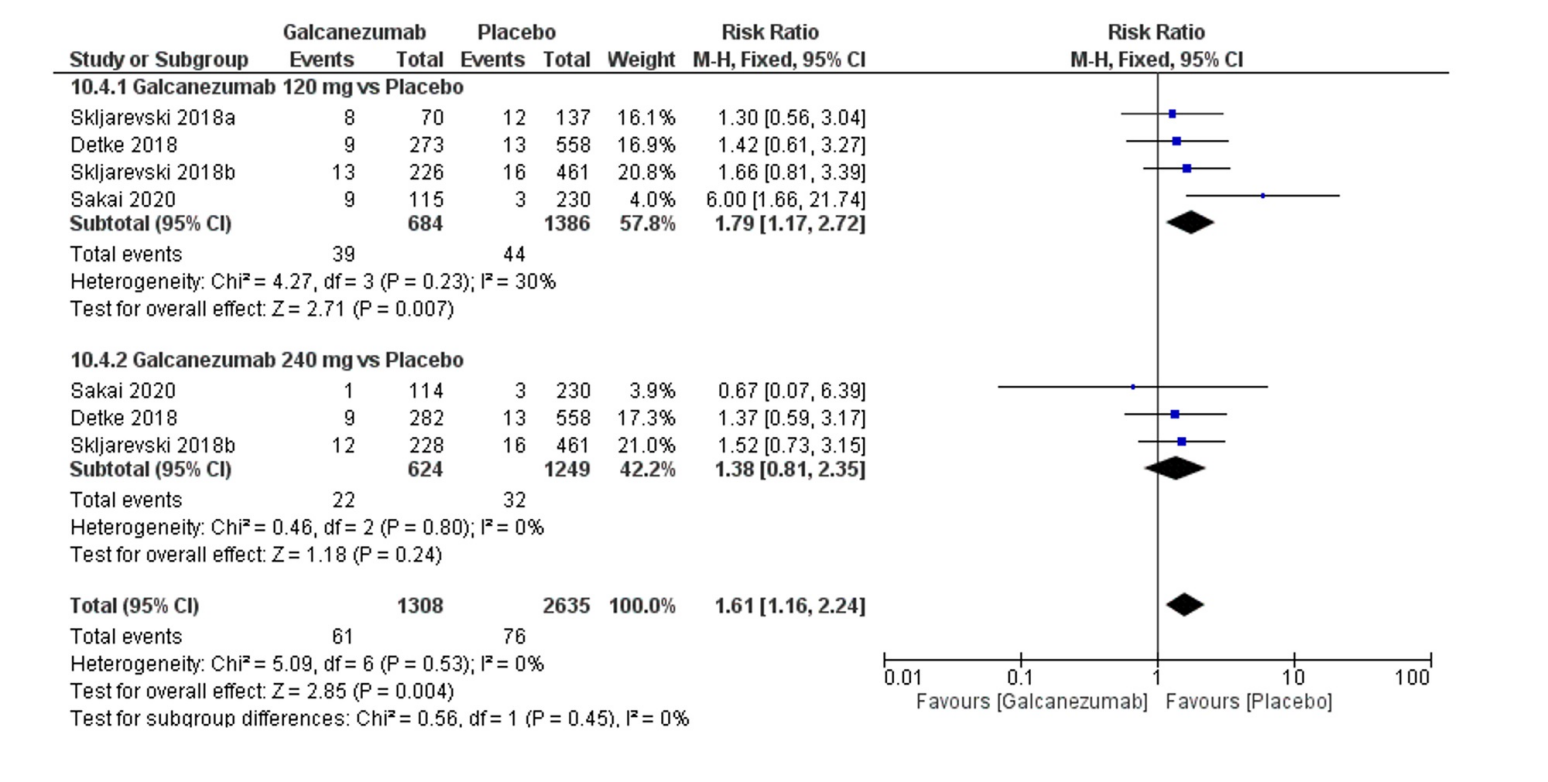
Forest plot showing the rate of upper respiratory tract infection between galcanezumab and placebo groups.

The overall effect size significantly showed a significantly higher frequency of URTI in the galcanezumab group (RR=1.61; 95% CI [1.16, 2.24]; p=0.004). Pooled results were homogenous (I^2^=0%; p=0.53), and the fixed-effects model was used (Figure 10). Subgroup analysis was performed according to the galcanezumab dose. For galcanezumab 120 mg versus placebo, the overall effect size revealed a significantly higher occurrence of URTI in the galcanezumab group (RR=1.79; 95% CI [1.17, 2.72]; p=0.007). Pooled results were homogeneous (I^2^=30%; p=0.23). For galcanezumab 240 mg versus placebo, the overall effect size did not differ between both groups (RR=1.38; 95% CI [0.81, 2.35]; p=0.24). Pooled results were homogeneous (I^2^=0%; p=0.80).

## Discussion

Our analysis found that galcanezumab was highly effective in the management of migraine attacks. Specifically, galcanezumab succeeded in decreasing monthly MHDs and monthly MHDs with acute medication use. Overall, when compared to placebo, our results revealed that both doses of galcanezumab provided nearly equal therapeutic efficacy for most outcomes, except for MSQ RF-R and MIDAS scores where galcanezumab 240 mg showed a significantly higher efficacy when compared with galcanezumab 120 mg. With regard to side effects, the rates of injection-site pain and nasopharyngitis did not substantially differ between galcanezumab (inclusive of 120 mg and 240 mg) and placebo groups. Nonetheless, when compared to placebo, galcanezumab 120 mg, but not galcanezumab 240 mg, substantially correlated with a higher rate of URTI.

The favorable efficacy of galcanezumab for the management of migraine is somehow anticipated, as galcanezumab has been depicted to be effective in managing other neurological disorders. In a recent review, galcanezumab has demonstrated promising results for both prevention and treatment of cluster headache [6,15,16]. Nonetheless, when administered to patients with osteoarthritis, galcanezumab failed to reduce signs and symptoms in patients with knee osteoarthritis [17].

Similar drugs of the same anti-CGRP monoclonal antibodies have been previously tried for migraine and reported encouraging results. For example, erenumab proved to be effective in the prevention and treatment of migraine [18]. A systematic review and meta-analysis of five randomized placebo-controlled trials revealed the superiority of erenumab over placebo in reducing the monthly MHDs and migraine-specific medication days [19]. Fremanezumab is a humanized monoclonal antibody that targets the CGRP receptor. Fremanezumab showed promising results in the treatment and prevention of migraine, with a very low incidence of side effects [20]. Both fremanezumab and erenumab could advantageously convert patients from chronic migraine status to episodic migraine status [21]. No trials till now have yet compared fremanezumab and galcanezumab to determine which drug is more effective and safer.

Generally, common side effects of anti-CGRP monoclonal antibodies include URTI, nasopharyngitis, urinary tract infection, and injection-site pain. Deng et al. [22] conducted a meta-analysis of 11 randomized placebo-controlled trials comparing anti-CGRP monoclonal antibodies versus placebo. The authors revealed that galcanezumab, fremanezumab, and erenumab significantly resulted in reduction of MHDs and acute migraine-specific medication days, in addition to an enhancement in 50% responder rate. Moreover, the adverse events and treatment discontinuation frequencies secondary to adverse events were not considerably dissimilar between the anti-CGRP monoclonal antibodies and placebo groups. In subgroup analysis, comparable efficacy and tolerability outcomes were achieved for galcanezumab, fremanezumab, and erenumab. Similar findings were reciprocated in other meta-analyses by Zhu et al. [23] and Xu et al. [24]. In the literature, various doses of galcanezumab have been used, ranging from as low as 5 mg to as high as 300 mg. The optimal dose that yields maximum efficacy and minimum adverse events is yet to be determined.

Our study has several strengths. The large number of included trials is the main strength of our study when compared to previous meta-analysis studies [25-27]. We only included randomized placebo-controlled clinical trials to ensure high-quality evidence. Moreover, we performed subgroup analysis according to the two most commonly used galcanezumab doses (120 mg and 240 mg) and excluded the others to ensure consistency with regard to drug dosing. Whenever heterogeneity existed during meta-analysis, we used the leave-one-out method to resolve the heterogeneity. Nonetheless, our study is not without limitations. The vast majority of studies had an unclear risk of bias regarding two important domains: allocation concealment and blinding of outcome assessment. This observation could negatively impact the quality of the evaluated outcomes. Moreover, some of the reported endpoints revealed significant heterogeneity, which could be ascribed to the varying degrees of migraine severity and duration of treatment. Lastly, not all studies adequately reported our prespecified side effects.

As it stands now, galcanezumab (120 mg and 240 mg) appears to be clinically safe and effective in the management of patients with migraine. Nonetheless, future research directions should be geared toward determining the optimal dose of galcanezumab for the management of patients with migraine. Moreover, head-to-head comparative studies between galcanezumab and other related anti-CGRP receptor monoclonal antibodies are warranted.

## Conclusions

In summary, this systematic review and meta-analysis examined the efficacy of galcanezumab (120 mg and 240 mg) versus placebo in patients with migraine. Our findings showed that galcanezumab (120 mg or 240 mg) was superior to placebo in reducing the number of MHDs and MHDs with acute medication use. Moreover, galcanezumab treatment significantly correlated with improved PGI-S, MSQ RF-R, and MIDAS scores. Overall, the rates of side effects did not substantially differ between galcanezumab and placebo groups.
